# Disseminated Cutaneous Leishmaniasis in a Patient Infected by *Leishmania panamensis*

**DOI:** 10.4269/ajtmh.18-0843

**Published:** 2019-03

**Authors:** Juan Carlos Cataño, Miguel Alejandro Pinzón

**Affiliations:** 1 Infectious Diseases Section, University of Antioquia Medical School, Medellin, Colombia;; 2 Infectious Diseases Section, Medellin clinic, Medellin, Colombia

**Figure 1.**
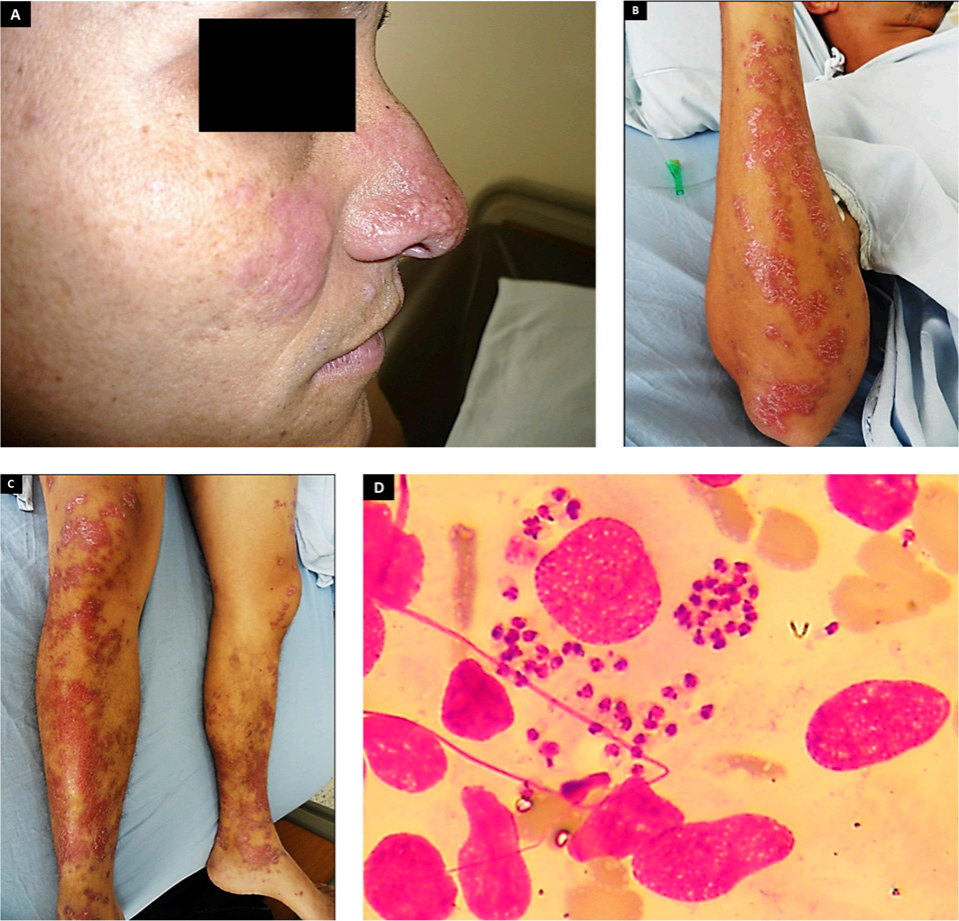
Multiple and disseminated erythematous desquamative plaques, which coalesce with a psoriasiform appearance, affecting face (**A**), arms (**B**), and legs (**C**). (**D**) Histopathology of skin biopsy showing multiple *Leishmania panamensis* amastigotes. This figure appears in color at www.ajtmh.org.

A 32-year-old injection drug user, with human immunodeficiency virus (HIV) infection diagnosed since 2000, who is intermittently adherent to antiretroviral therapy, presented to an infectious diseases outpatient clinic, with a 5-year history of multiple disseminated non-pruritic skin lesions, which started with an erythematous rash on his left hand, which within the next years spread to the face, hands, and legs bilaterally, associated with subjective fever and malaise, but no mucosal compromise or other significant symptoms related. On examination, he appeared chronically ill and wasted (lost 10 kg over 2 years). Vital signs showed a blood pressure of 100/60 mm Hg, pulse 120/minute, and temperature 38.2°C. There were no mouth lesions, and chest auscultation was normal; on abdominal examination, the liver was enlarged (span, 14 cm) but not tender, and cervical and axillary lymphadenopathies were found. On skin, multiple and disseminated erythematous desquamative plaques, which coalesce with a psoriasiform appearance, were found in several anatomic surfaces, but without signs of superinfection (Figure 1A–C). The remainder of the physical examination was normal. Laboratory data showed white blood cells 13.5 cells/mL (15.7% eosinophils), hemoglobin 12.4 mg/dL, platelets 175,000/mL, creatinine 0.7 mg/dL, and CD4 count 17 cells/uL. Several skin biopsies were performed, showing multiple *Leishmania panamensis* amastigotes (Figure 1D), which was confirmed by specific polymerase chain reaction. The patient was started on amphotericin B deoxycholate, showing a rapid improvement of lesions, but leaving severe esthetic sequels.

Leishmaniasis is a vector-borne chronic infectious disease, transmitted via the bite of sandflies belonging to either *Phlebotomus* spp. (in Europe, North Africa, the Middle East, and Asia) or *Lutzomyia* spp. (from the southern United States to northern Argentina), and caused by a group of protozoan parasites of the genus *Leishmania*.^1^ Cutaneous leishmaniasis can have a number of particular features in individuals with immunosuppression, especially if severe, including parasite dissemination, clinical polymorphism with atypical and often more severe clinical forms, and even visceralization.^2^ The best diagnostic approach is the combination of parasitological and serological or molecular methods. Amphotericin B is the drug of choice, but treatment failure and relapse rates are particularly high in cases of HIV coinfection, despite the initiation of antiretroviral treatment.^3^ Primary prophylaxis is not recommended, but secondary prophylaxis is recommended when the patient is immunosuppressed.
